# Downregulation of miRNA-26a by HIV-1 Enhances CD59 Expression and Packaging, Impacting Virus Susceptibility to Antibody-Dependent Complement-Mediated Lysis

**DOI:** 10.3390/v16071076

**Published:** 2024-07-04

**Authors:** Nicolas Bellini, Chengyu Ye, Oluwaseun Ajibola, Thomas T. Murooka, Robert Lodge, Éric A. Cohen

**Affiliations:** 1 Laboratory of Human Retrovirology, Institut de Recherches Cliniques de Montréal, Montreal, QC H2W 1R7, Canada; nicolas.bellini@ircm.qc.ca (N.B.); chengyu.ye@mail.mcgill.ca (C.Y.); robert.lodge@ircm.qc.ca (R.L.); 2 Department of Microbiology, Infectious Diseases and Immunology, Faculty of Medicine, Université de Montréal, Montreal, QC H3C 3J7, Canada; 3 Department of Immunology, Rady Faculty of Health Sciences, University of Manitoba, Winnipeg, MB R3E 0T5, Canada; oluwaseun.ajibola@umanitoba.ca (O.A.); thomas.murooka@umanitoba.ca (T.T.M.)

**Keywords:** CD59, HIV-1, complement-mediated lysis, miRNA-26a, innate immunity, viral evasion

## Abstract

MicroRNAs (miRNAs) play important roles in the control of HIV-1 infection. Here, we performed RNA-seq profiling of miRNAs and mRNAs expressed in CD4^+^ T lymphocytes upon HIV-1 infection. Our results reveal significant alterations in miRNA and mRNA expression profiles in infected relative to uninfected cells. One of the miRNAs markedly downregulated in infected cells is miRNA-26a. Among the putative targets of miRNA-26a are CD59 receptor transcripts, which are significantly upregulated in infected CD4^+^ T cells. The addition of miRNA-26a mimics to CD4^+^ T cells reduces CD59 at both the mRNA and surface protein levels, validating CD59 as a miRNA-26a target. Consistent with the reported inhibitory role of CD59 in complement-mediated lysis (CML), knocking out CD59 in CD4^+^ T cells renders both HIV-1-infected cells and progeny virions more prone to antibody-dependent CML (ADCML). The addition of miRNA-26a mimics to infected cells leads to enhanced sensitivity of progeny virions to ADCML, a condition linked to a reduction in CD59 packaging into released virions. Lastly, HIV-1-mediated downregulation of miRNA-26a expression is shown to be dependent on integrated HIV-1 expression but does not involve viral accessory proteins. Overall, these results highlight a novel mechanism by which HIV-1 limits ADCML by upregulating CD59 expression via miRNA-26a downmodulation.

## 1. Introduction

The identification of host and viral determinants that govern human immunodeficiency virus type 1 (HIV-1) transmission and infection is critical for the development of effective targeted prevention and therapeutic approaches. Following infection by a transmitter founder (T/F) virus, a humoral response is rapidly established with mostly non-neutralizing antibodies (Abs) being directed against HIV-1 envelope glycoproteins (Env, gp120 and gp41) [1,2,3]. Neutralizing Abs specific for the T/F virus then promptly emerge; however, these autologous neutralizing Abs, which target highly variable regions of the Env, rapidly select virus-escape mutants that in turn induce new Ab specificities [4,5,6]. The ensuing Ab-virus “arms race” leads, after years of infection, to the induction of broadly neutralizing antibodies (bNAbs) in only 10–25% of chronically infected individuals, which target highly conserved Env regions and have higher and broader neutralizing potency [7]. While the host humoral response exerts immune pressure directly on replicating viruses, the binding of Abs to Env epitopes can also lead to activation of the classical complement pathway, a process that results in the lysis of infected cells and virions [8].

Complement can be activated by three canonical mechanisms, designated as the classical, alternative and lectin pathways [9]. Ultimately, all three pathways lead to the formation of the membrane attack complex (MAC), as well as the formation of anaphylatoxins, which contribute to inflammation by attracting leukocytes to the site of infection [10]. Among the complement-regulatory proteins that are present at the surface of human cells is CD59, a glycosyl-phosphatidylinositol (GPI)-linked cellular receptor, which prevents the polymerization of component 9 (C9) and thus inhibits the formation of MAC [11,12]. Interestingly, HIV-1 has been shown to incorporate CD59 and other complement-regulatory proteins, such as CD55, into its viral envelope during the budding process, a condition that confers protection against complement-mediated lysis (CML) [13,14]. While antibody-dependent mediated complement activity directed against HIV-1 virions is detected during acute infection [15,16]; it remains unclear whether HIV-1 has evolved to limit this host antiviral response.

MicroRNAs (miRNAs) are small single-stranded non-coding RNA molecules (containing about 22 nucleotides) encoded by plants, mammals and even viruses. They are involved in the post-transcriptional regulation of gene expression and RNA silencing [17]. MiRNAs work by base pairing with complementary sequences in the target mRNA, primarily in the 3′ untranslated region (UTR). As a result, the targeted mRNA is silenced by one or more mechanisms, including mRNA cleavage and mRNA translation blockage by ribosomes [18]. Host miRNAs have been shown to play a key role during HIV-1 infection by regulating cellular factors that govern the susceptibility or the resistance of target cells to viral infection [19]. For instance, the upregulation of miRNAs-221/222 inhibits HIV-1 infection by downregulating the virus primary CD4 receptor in lymphocytes, as well as in monocyte-derived macrophages (MDMs) [20,21]. More recently, we provided evidence that the p53-regulated miRNA-103 participates in the downmodulation of CCR5 in macrophages [22], as well as during different stages of CD4^+^ T cell differentiation [23], thus regulating the process of HIV-1 entry into these cells. MiRNAs can also regulate HIV-1 infection at post-entry steps as shown for miRNA-181, which targets the restriction factor SAMHD1 in monocytes [24]. This is also the case for miRNA-155, which promotes HIV-1 latency by targeting the E3 ubiquitin ligase TRIM32 that activates NF-κB [25].

In the context of HIV-1 infection, a large number of host factor-targeting miRNAs identified are upregulated and modulate the permissiveness of target cells to productive infection by downregulating the expression of HIV-1-dependency or restriction factors. So far, several miRNA/mRNA transcriptomic studies have been previously performed on HIV-1 infected CD4^+^ T cells [26,27,28,29,30]. However, most of these studies were not conducted on isolated infected cells and not in parallel to miRNA profiling in the same samples. In this study, we performed a global transcriptomic analysis of HIV-1 infected primary CD4^+^ T cells and compared their miRNA and mRNA profiles based on the same isolated HIV-1 infected cell samples and compared them with infected cells. Our results reveal the significant differential expression of both miRNAs and mRNAs in infected relative to uninfected cells. Among the miRNAs analyzed, we show that miRNA-26a is downregulated in infected cells and further identify the MAC-regulatory CD59 receptor as a target of this miRNA, which indeed is found to be upregulated. Accordingly, we document that enhancing miRNA-26a in HIV-1-infected cells reduces CD59 expression and packaging into virions, a condition that impacts their susceptibility to antibody-dependent complement-mediated lysis (ADCML). These results underscore the role of miRNA-26a in regulating CD59 expression levels in CD4^+^ T cells and highlight how the modulation of miRNA-26a may affect HIV-1 susceptibility to ADCML by regulating the extent of CD59 packaging into HIV-1 virions.

## 2. Methods

### 2.1. Human Subjects, Informed Consent Statement and Institutional Review Board Statement

Peripheral blood samples and leukaphereses were obtained from HIV-1- and HCV-seronegative adults (of either sex). All participants had given written informed consent in accordance with the Declaration of Helsinki under research protocols approved by the research ethics review board of the Institut de recherches cliniques de Montréal (IRCM).

### 2.2. Cells

Human embryonic kidney (HEK)-293T cells were obtained from ATCC and were maintained in Dulbecco’s modified Eagle’s medium (DMEM) (Wisent, St-Bruno, QC, Canada) supplemented with 10% heat inactivated fetal bovine serum (FBS) (Wisent).

CEM.NKR-CCR5 cells were obtained from the NIH HIV Reagent Program (ATCC, BEI Resources, Manassas, VA, USA) (designated as CEM-CCR5 throughout the text) and were maintained in Roswell Park Memorial Institute (RPMI) 1640 medium (Wisent) supplemented with 10% FBS.

CD4^+^ T cells were isolated from PBMCs via negative depletion using the human CD4^+^ Isolation Kit II (Miltenyi, San Diego, CA, USA, #130-096-533) according to manufacturer’s instructions. Cells were cultured in RPMI-1640 medium containing 10% FBS. Activated CD4^+^ T cells were generated by co-stimulation with 10 μg/mL of anti-CD3 (Biolegend, San Diego, CA, USA, #300438) and 2 μg/mL of anti-CD28 (Biolegend, #302934) Abs and cultured in the presence of 100 U/mL of IL-2 (Peprotech, Cranbury, NJ, USA, #200-02-100UG) for 4 days.

### 2.3. Proviral DNA Constructs

The CCR5-tropic NL4.3-ADA-GFP-IRES-NEF fully replicative proviral construct (named NL4.3-ADA-GFP throughout the text), which encodes all accessory proteins and its isogenic derivatives, Vpr-deficient (ΔVpr), Vpu-deficient (ΔVpu), Nef-deficient (ΔNef), Nef- and Vpu-deficient (ΔNefΔVpu), were previously described [31,32,33]. The Vif-deficient (ΔVif) isogenic derivative was generated by inserting an SpeI (NEB, Ipswich, MA, USA, #R3133L) to SalI (NEB, #R3138L) fragment encompassing a *vif* deletion from the NL–PI*vif*-construct [34] into NL4.3-ADA-GFP.

The E2-Crimson-EF1alpha-ZsGreen DNA insert was amplified from the Hi.Fate latency plasmid [35] using primers OligoA and OligoB (see Table S1) via PCR and inserted into the R5-tropic ‘HIV-GFP’ proviral vector [36] using unique restriction enzyme sites, XmaI (NEB, #R0180S) and MluI (NEB, #R0198S). The P2A sequence from Porcine Teschovirus-1 [37] was generated using custom oligonucleotides and inserted into the MluI site between the *Nef* and *E2-Crimson* gene locus. The resulting plasmid, termed ‘HIV Nef-2A-CRIMZs’, was sequenced on both strands before transfection into HEK293T cells.

### 2.4. Virus Production and Infection

NL4.3-ADA-GFP-derived viruses were generated by co-transfecting the corresponding proviral constructs with pSVCMV-VSV-G [38] (except in the case of viruses used in RNAseq profiling studies) in 5 × 10^6^ HEK-293T cells using Lipofectamine 3000 (Invitrogen, ThermoFisher Scientific, Waltham, MA, USA) according to manufacturer’s instructions. Virus-containing supernatants were recovered 48 h post-transfection, cleared of cells via centrifugation at low speed and filtered, and virus pellets were recovered following ultracentrifugation (35,000 rpm for 3 h) on a 20% sucrose cushion. Viruses were resuspended in DMEM (with FBS), and aliquots were kept at −80 °C. The multiplicity of infection (MOI) was determined using the CEM-CCR5 cell line.

Activated primary CD4^+^ T cells were infected with NL4.3-ADA-GFP at an MOI of 2 in the case of the RNA-seq profiling studies or pseudotyped with VSV-G and infected at an MOI of 1 in all other cases (WT or mutant NL4.3-ADA-GFP infection). Infections were performed via spinoculation (centrifugation at 1200× *g* at 22 °C for 2 h). Cells were washed 6 h after viral adsorption with Phosphate Buffered Saline (PBS), and the infection efficiency was determined 48 h post-infection through measurements of the frequency of GFP-positive (GFP-pos) cells via flow cytometry. In some cases, primary CD4^+^ T cells were pre-treated with efavirenz (EFV, 10 μM, Sigma-Aldrich, St. Louis, MO, USA, #SML0536) or raltegravir (RAL, 20 μM, Santa Cruz Biotechnology, Dallas, TX, USA, #sc-364600) overnight and replenished in new media accordingly during the infection.

### 2.5. Antibodies and Flow Cytometry

The following Abs were used in flow cytometry assays: BV421 anti-human CCR5 (Biolegend, #359118), PE/Cyanine7 anti-human CD4 (Biolegend, #317414) and PE anti-human CD59 (Miltenyi, #130-120-048), as well as its isotype control (Miltenyi, #130-113-438). For surface staining, fluorochrome-labeled Abs were added directly to the CD4^+^ T cells at a final dilution 1:50 (Miltenyi) or 1:150 (Biolegend), incubated for 45 min on ice, washed twice in FACS buffer (PBS, 1% of FBS) and fixed with 4% paraformaldehyde in PBS. Total CD59 (surface + intracellular proteins) was measured by fixing and permeabilizing cells using the Cytofix/Cytoperm kit (BD Biosciences, Franklin Lakes, NJ, USA, #554714) as per the manufacturer’s instructions and staining with anti-CD59 Abs. Cells were resuspended in PBS-EDTA for flow cytometry analyses. Flow cytometry and cell sorting were performed on a FACSAria III (BD Biosciences) or CyAn (Beckman Coulter, Mississauga, ON, Canada) cytometer equipped with appropriate lasers. Acquired data were analyzed with FlowJo 10.9 software (BD Biosciences).

### 2.6. Transfection of Primary CD4^+^ T Cells

Primary CD4^+^ T cells were transfected with 340 pmol of either negative control RNA (Qiagen, Germantown, MD, USA, #YM00479902-ADB), mimics of miRNA-21 (Qiagen, #YM00473093-ADB), mimics of miRNA-26a (Qiagen, #YM00471417-ADB) or mimics of miRNA-29a (Qiagen, #YM00472650-ADB) using nucleofection (Lonza, Walkersville, MD, USA) according to the manufacturer’s instructions and cultured for 48 h in 48-well plates in basal medium (RPMI-1640) supplemented with IL-2 (100 U/mL). The negative control and all the miRCURY locked nucleic acid (LNA) mimics were fluorescein amidites (FAM)-labeled (see Table S1).

### 2.7. RNA Extraction, Reverse-Transcription and Real-Time qPCR Analyses

CD4^+^ T cells infected with the different viruses described above were sorted using an Influx cell sorter (BD Biosciences) and recovered in RLT buffer. Total cellular RNAs were extracted using RNeasy RNA extraction columns (Qiagen) according to the manufacturer’s instructions. Total RNAs (100 ng) were reversed transcribed using SuperScript IV reverse transcriptase (Invitrogen) with poly(dT) and specific loop primers (see Table S1) for the appropriate miRNAs using the two-tailed real-time qPCR method [39]. For real-time qPCR, cDNA and appropriate primers (see Table S1) were added to SYBR green select master mix (Applied Biosystems, ThermoFisher Scientific) in 96-well plates and run on a ViiA96 thermocycler (ThermoFisher Scientific) with the following cycling conditions: 50 °C for 2 min, 95 °C for 5 min, and 40 cycles at 95 °C for 20 s and at 60 °C for 40 s. Glyceraldehyde 3-phosphate dehydrogenase (GAPDH) or small nuclear RNA (snRNA) U6 were used as endogenous controls (see primers in Table S1), and ΔΔCT variations were calculated.

### 2.8. CD59 3′ UTR Validation Assay

To generate pMIR-REPORT-CD59-Luc, a PCR was performed on the cDNA of primary CD4^+^ T cells using pfu DNA polymerase (see primers in Table S1), and different fragments of the 3′ UTR of CD59 were inserted into the SpeI and MluI sites of pMIR-REPORT-Luc. Site-directed mutagenesis of the 3′ UTR of CD59 was performed via PCR to generate mutations (see Mut (mutation) primers in Table S1) and cloned into pMIR-REPORT-Luc using the same strategy described above. Fragment A corresponds to nucleotides 449-657 of the 3′ UTR of CD59 (based on accession number #NM_000611.6), while fragment B corresponds to nucleotides 666-878. Finally, the AB fragment corresponds to nucleotides 449-878 of the 3′ UTR of CD59 and was generated using the primers indicated in Table S1.

For the MIR-REPORT assay, HEK-293T cells were co-transfected with pGL4.70Actin1.2(8) [40] and either one of the plasmids derived from pMIR-REPORT-CD59-Luc. Cells were further treated with either control or mimics (67 pmol) of miRNA-26a at 24 h post-transfection and lysed after another 24 h. Luciferase activity was measured using the Dual-Glo Luciferase Assay System (Promega, Madison, WI, USA) on a GloMax luminometer (Promega).

### 2.9. Generation of the CEM-CCR5 CD59 Knock-Out Cell Line

To generate CD59KO CEM-CCR5 cell lines, a guide sequence (see Table S1) previously described as targeting CD59 [41] was inserted into the lentiCRISPR v2 vector [42]. Lentiviruses were produced via the transfection of this vector in combination with pSVCMV-VSV-G in HEK-293T cells. Control lentiviruses were also produced using the lentiCRISPR v2 without the single guide RNA (sgRNA). CEM-CCR5 cells were transduced with either the control or sgRNA-expressing lentiviruses, and transduced cells were selected with puromycin (2 μg/mL). Individual clones (CEM-CD59_KO) were obtained via single-cell sorting.

### 2.10. Purification of Antibodies

Abs from the sera of viremic individuals were purified using protein G Dynabeads (Life Technologies, ThermoFisher Scientific, #10003D), according to the manufacturer’s instructions. The quantity of Abs obtained was evaluated by performing a protein assay (Bio-Rad, Hercules, CA, USA).

### 2.11. Antibody-Dependent Complement-Mediated Lysis Assay

A mix of sera from three to five healthy donors was used as a source of complement.

The virion ADCML assay was performed as previously described [15] with some modifications. HIV-1 virions were produced from primary T lymphocytes, CEM-CD59-control or CEM-CD59_KO cells infected with NL4.3-ADA-GFP. Equal amounts of viruses (as determined via ELISA for HIV-1 p24 (XpressBio, Frederick, MD, USA, Cat#XB-1000) for CEM or HIV-1 genomic equivalents for primary T lymphocytes) were then incubated with 1 mg/mL of RNase A (Invitrogen, #12091021), the bNAb PGT121 (10 μg/mL, obtained from the NIH HIV Reagent Program) or the purified Abs from patient sera (final dilution 1:5) and with either normal human serum (NHS) or heat-inactivated human serum (HIHS, dilution 1:2) in RPMI-1640 for a total volume of 140 µL. The mixture was then incubated for 3 h at 37 °C prior to freezing at −20 °C. Following thawing, the samples were treated with RNase A (1 mg/mL) and DNase I (1 mg/mL) (Sigma-Aldrich, #11284932001) for 1 h at 37 °C and subsequently treated with proteinase K (1 mg/mL) (Invitrogen, #25530049) to remove RNase and DNase activity. Residual viral RNA in intact virions was extracted using the QIAamp Viral RNA Mini Kit (Qiagen) according to manufacturer’s instructions, reverse-transcribed and quantified via real-time PCR. Total HIV-1 RNA was quantified using a modified nested real-time PCR assay using Taq DNA polymerase (NEB) in a first PCR and the TaqMax Fast Advanced Master Mix (Applied Biosystems) in a second PCR as previously described [43] (see HIV-TOT primers in Table S1). The resulting HIV-1 genomic equivalents were determined using a standard curve. This standard curve was generated by extracting and amplifying DNA from serially diluted ACH-2 cells, which contain a single integrated copy of HIV-1 (NIH HIV Reagent Program). In each assay, samples were tested in triplicate.

For ADCML of cells, CEM-CD59-control or CEM-CD59_KO cells were infected with NL4.3-ADA-GFP at comparable infection rates and incubated in the presence of 50% NHS or HIHS, either with or without PGT121 (10 μM), overnight. To measure ADCML, cells were treated with the Zombie NIR Fixable Viability marker (1:150 in PBS; Biolegend, #423105) and analyzed via flow cytometry.

### 2.12. Virion Capture Assay

Virus capture assays were performed as previously described [44] with some modifications. Immunomagnetic bead-based virion capture was performed with 20 μL of protein G Dynabeads (Life Technologies; #10003D), which were incubated with 2 μL of mouse anti-CD59 (Biolegend, #304702) for 20 min at room temperature (RT) and then washed with PBS to remove unbound Ab. At the start of capture assays, virus input was normalized across all viruses tested, with inputs corresponding to equal virus volumes (100 μL) and the same infection rates. Virus samples were incubated with Ab-armed beads for 1 h at RT to allow for virus capture. Beads were then washed five times with PBS to extensively remove unbound virus particles. The bead-associated virus was then lysed with lysis buffer and quantified via p24 ELISA (XpressBio) following the manufacturer’s instructions. Background levels of virion capture were assessed by using an isotype control Ab (Biolegend, #401501). The level of background virus capture was subtracted from each data point.

### 2.13. RNA-Seq of Sorted HIV-1-Infected CD4^+^ T Cells for mRNA/miRNA Profiling and Analyses of Their Expression

Productively infected (GFP-positive) and uninfected CD4^+^ T cell populations were sorted using an Influx cell sorter (BD Biosciences) and directly recovered in RLT lysis buffer. The same samples were used for miRNA-seq and mRNA-seq using the Illumina TruSeq mRNA or Small RNA system (Illumina Technologies, San Diego, CA, USA) at the IRCM Molecular Biology and Functional Genomics Core Facility. Specific tagging was used to identify RNA from each blood donor. The resulting library was sequenced using a NovaSeq 6000 system (Illumina Technologies). Reads were trimmed with Cutadapt v2.5, aligned to the reference genome GRCh38 using STAR version 2.5.1b extracted using FeatureCounts v1.6.0, and specifically, miRNAs were quantified using mirdeep2 v2.0.1.1. Differential expression of miRNAs and mRNAs was assessed using adjusted *p* values computed using DESeq2 v1.24.0. Volcano plots were generated using GraphPad Prism9 (GraphPad Software, LLC, La Jolla, CA, USA). The online search for miRNA and mRNA pairing was performed using mirDIP (http://ophid.utoronto.ca/mirDIP/ (accessed on 26 January 2024)). Gene ontology analyses were performed using g:Profiler (https://biit.cs.ut.ee/gprofiler/gost (accessed on 26 January 2024)).

### 2.14. Quantification and Statistical Analyses

Statistical analyses were performed using GraphPad Prism (Version 8). Non-parametric Mann-Whitney’s U-tests (two-tailed) were used to compare ranks between two groups or more. The non-parametric Kruskal Wallis test was used when indicated. A *p* value of less than 0.05 was considered statistically significant. *, ** and *** signify <0.05, <0.01 and <0.001, respectively, with ns as not significant.

## 3. Results

### 3.1. HIV-1 Alters the miRNA and mRNA Expression Profiles during Infection of CD4^+^ T Cells

**Figure 1:**
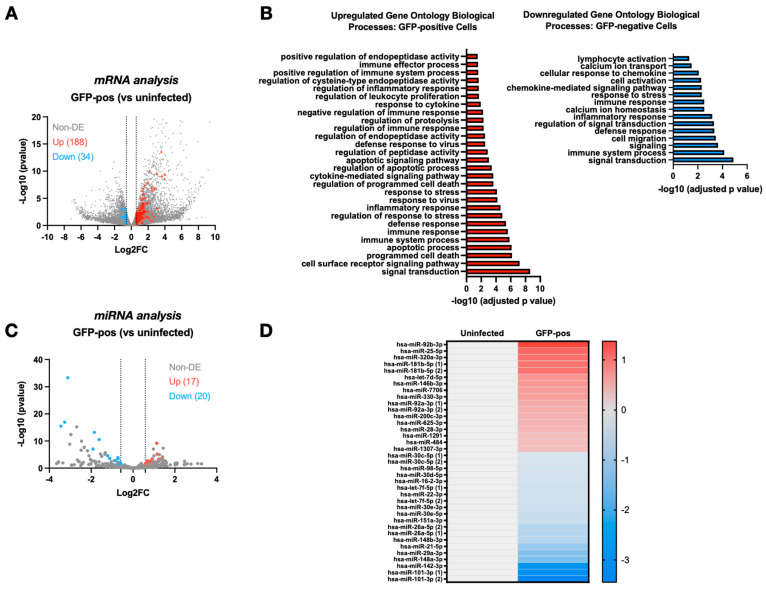
RNA-seq mRNA and miRNA profiles in infected CD4^+^ T cells compared to uninfected cells. (**A**) Volcano plot representing the differential expression (FC = fold change) of mRNAs in infected (GFP-positive, GFP-pos) CD4^+^ T cells as compared to uninfected cells. The mRNAs with a log2FC of >0.585 and a DeSeq2 base mean > 100 are in red, while those with a log2FC < −0.585 are in blue. (**B**) Graphs representing the upregulated (left panel) or downregulated (right panel) biological processes in infected (GFP-pos) as compared to uninfected cells as determined using g:Profiler (https://biit.cs.ut.ee/gprofiler/gost (accessed on 26 January 2024)). (**C**) Volcano plot representing the differential expression of miRNAs in infected (GFP-pos) CD4^+^ T cells as compared to uninfected cells. Dots of upregulated miRNAs with a log2FC of >0.585 and a DeSeq2 base mean > 100 are in red, while those downregulated with a log2FC < −0.585 are in blue. (**D**) The heatmap depicts the 37 miRNAs specifically dysregulated in the GFP-pos population detected in this study. The scale bar is in log2FC. MiRNAs with forms (1) and (2) are identical miRNAs produced by two distinct loci. NDE stands for non-differentially expressed. See also Figures S1 and S2.

In order to identify miRNAs and mRNAs that are modulated upon HIV-1 infection, CD4^+^ T lymphocytes were isolated from the peripheral blood mononuclear cells (PBMCs) of three blood donors and infected for 48 h with green fluorescent protein (GFP)-marked HIV-1 (CCR5-tropic NL4.3-ADA-GFP). Infected cells (GFP-positive) were then enriched via flow cytometry sorting (Figure S1), and total RNAs were extracted. RNAs from uninfected CD4^+^ T cells were used as a control. MiRNA and mRNA expression profiles in uninfected and GFP-positive CD4^+^ T lymphocytes were then assessed via next-generation RNA-seq (Figure 1). MiRNAs or mRNAs with a log2 fold-change (FC) of >0.585 (FC = ~1.5) and a computed (using DeSeq2) base mean >100 were deemed upregulated whereas those with a log2FC < −0.585 were considered downregulated. GFP-positive infected cells displayed a distinct profile of cellular mRNAs as compared to uninfected cells, with 188 upregulated and 34 downregulated genes (Figure 1A and Figure S2). Gene ontology analyses (using g:Profiler, https://biit.cs.ut.ee/gprofiler/gost (accessed on 26 January 2024)) of the most differentially expressed genes (both upregulated and downregulated) revealed a predominance of genes involved in signal transduction, immune system processes, immune and defense response, inflammatory response, programmed cell death and responses to stress and virus (Figure 1B). Interestingly, comparative analyses of the miRNA-seq profiles of GFP-positive cells with that of uninfected cells identified 17 upregulated and 20 downregulated miRNAs (Figure 1C), indicating that the productive infection of CD4^+^ T cells also impacted the cellular miRNA expression profile. Our analyses identified several miRNAs for which expression was previously reported to be modulated upon HIV-1 infection (Figure 1D). For instance, miRNA-181, a miRNA that is known to target the restriction factor SAMHD1, is upregulated in infected cells [24]. Similarly, miRNA-25, a miRNA that we recently found to target peroxisomes components [45], as well as the E3 ubiquitin ligase MARCH1 [46], is similarly increased in infected CD4^+^ T cells. In contrast, we found that some miRNAs, such as miRNA-16, were downregulated in GFP-positive CD4^+^ T cells (Figure 1D). MiRNA-16 was previously reported to target Pur-α, a DNA- and RNA-binding protein that promotes Tat-induced transactivation [47].

### 3.2. Analyses of mRNA and miRNA Profiles in Infected CD4^+^ T Cells Link CD59 Upregulation to a Decrease in Putative Targeting miRNAs

**Figure 2:**
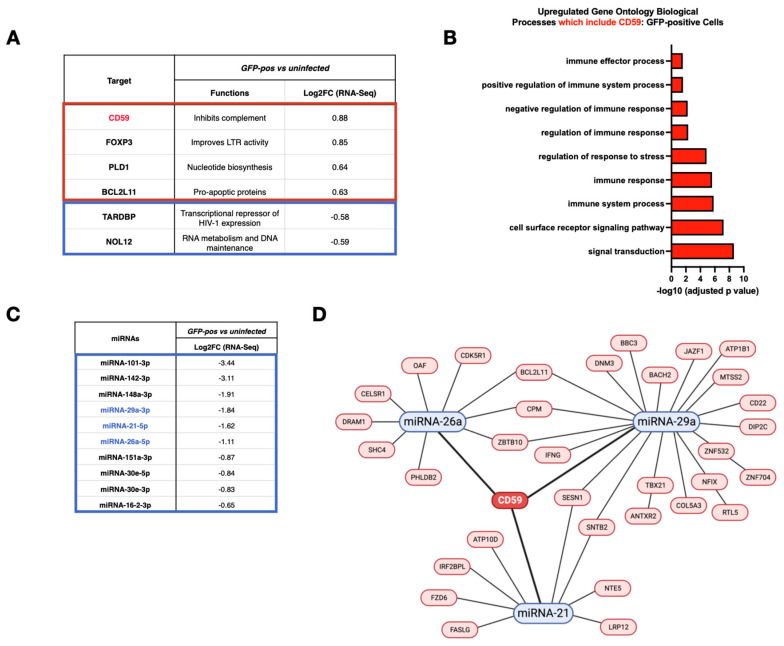
CD59 is predicted to be regulated, in infected cells, by three miRNAs. (**A**) Example of genes for which expression is dysregulated in infected cells (GFP-pos) compared to uninfected cells and that are relevant in the context of HIV-1 infection based on the current literature. The FC values indicated correspond to the values obtained based on the RNA-seq analysis. (**B**) Graph representing the upregulated gene ontology pathways (from Figure 1B, left panel), which include CD59. (**C**) Shown are the 10 most downregulated miRNAs in GFP-pos cells compared to uninfected, ranked from most to least decreased. The FC values indicated correspond to the values based on the RNA-seq analysis. (**D**) MiRNAs-21, -26a and 29a potentially target many dysregulated genes in GFP-pos cells, including CD59. Cross-referencing of miRNA and transcriptomic RNA data was performed using online databases (mirDIP, http://ophid.utoronto.ca/mirDIP/ (accessed on 26 January 2024)).

**Figure 3:**
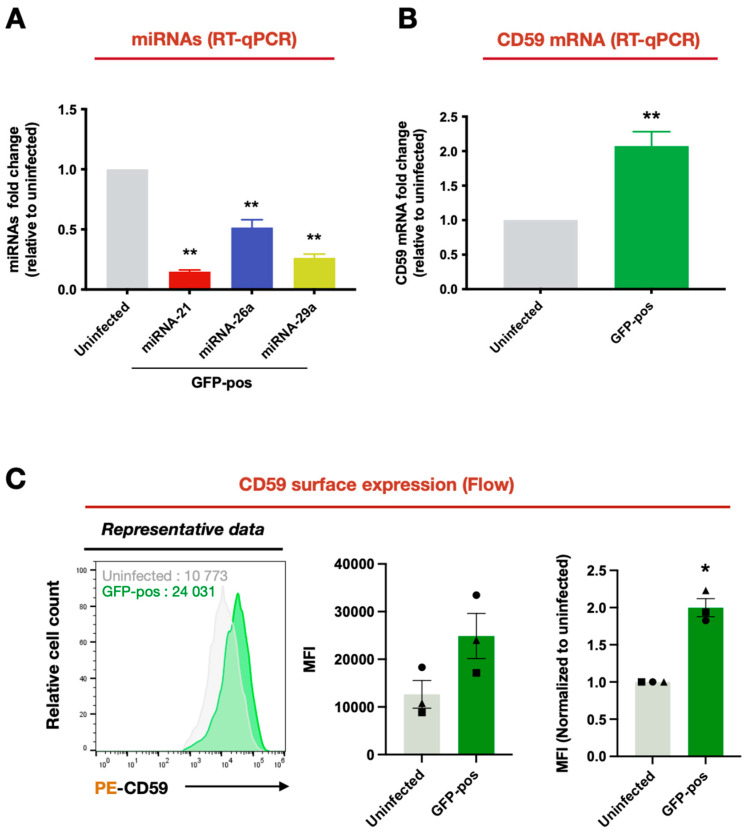
Validation of the differential expression of predicted CD59-targeting miRNAs (miRNAs-21, miRNA-26a and miRNA-29a) and CD59 upon HIV-1 infection. Expression levels of miRNAs-21, -26a and -29a (**A**) and CD59 mRNA (**B**) in uninfected and productively infected (GFP-pos) CD4^+^ T cells (n = 6) were measured via real-time qPCR. Shown are the mean fold-changes compared to uninfected cells (in gray, which is set at 1). Error bars represent the standard error of the mean (SEM). Statistical significance was determined using the nonparametric Mann–Whitney’s test, values: ** *p* < 0.01. (**C**) CD59 cell-surface expression was evaluated via flow cytometry, and the mean fluorescent intensity (MFI) was compared between uninfected and infected CD4^+^ T cells (GFP-positive) obtained from healthy blood donors. The left panel is representative data from cells from one donor, the middle panel is the MFI of cells from three donors and the right panel is normalized data (as compared to uninfected cells). Error bars represent the SEM (n = 3). Mann–Whitney’s test, values: * *p* = 0.05.

Among the 34 genes for which mRNA levels were downregulated in GFP-positive compared to uninfected cells, we identified several genes potentially relevant to HIV-1 infection based on the reported literature (Figure 2A). For example, Transactive Response DNA-binding protein (*TARDBP/TDP-43*) encodes a protein that regulates cell permissivity to HIV-1 infection by acting as a transcriptional repressor of HIV-1 gene expression through histone deacetylase 6 (HDAC6) [48], which is also a negative regulator of the APOBEC3G restriction factor [49] (log2FC = −0.58). *NOL12* encodes an RNA-binding protein that plays an important role in repairing DNA damage pathways and maintaining genome integrity, although its effect on HIV-1 infection remains unclear [50] (log2FC = −0.59). On the other hand, among the 188 genes for which mRNA levels were upregulated, we also identified cellular genes relevant to HIV-1 infection. For instance, *BCL2L11* (or *BIM*), which encodes Bcl2, a regulator of pro-apoptotic signaling in HIV-1 infection [51] (log2FC = 0.63), *PLD1*, which encodes Phospholipase D1, an enzyme that links T cell activation signals to increased cell permissivity to HIV-1 infection [52] (log2FC = 0.64), and *FOXP3*, which encodes a marker of regulatory CD4^+^ T cells [53] (log2FC = 0.85) all displayed an upregulated expression in GFP-positive cells (Figure 2A). Noticeably, *CD59,* which encodes a GPI-linked membrane protein receptor that has a key inhibitory role in regulating complement activation [11,12], was also found to show a significant upregulation of its expression in GFP-positive cells (log2FC = 0.88) (Figure 2A). Indeed, CD59 was also found to be among the genes in nine of the upregulated ontology pathways that we identified (Figure 1B, left panel, and Figure 2B). To assess whether the upregulation of CD59 mRNA was linked to the downregulation of specific miRNAs, we first identified the miRNAs that were the most downregulated in GFP-positive cells (Figure 2C). Online database-directed analyses (mirDIP, http://ophid.utoronto.ca/mirDIP/ (accessed on 26 January 2024)) singled-out three potential miRNAs that could target CD59 mRNA (Figure 2C,D): miRNA-21 (log2FC = −1.62), miRNA-26a (log2FC = −1.11) and miRNA-29a (log2FC = −1.84). Further validation of the miRNA-seq data using real-time qPCR revealed that miRNAs-21, -26a and -29a are indeed decreased in HIV-1-infected primary CD4^+^ T cells as compared to uninfected cells (~6-, 2- and 4-fold less, *p* = 0.0003, *p* = 0.0484 and *p* = 0.0020, respectively) (Figure 3A). We also validated the mRNA-seq data and found that CD59 mRNA levels are indeed increased in GFP-positive relative to uninfected primary CD4^+^ T cells (FC = 2.1-fold, *p* = 0.0022) (Figure 3B). Furthermore, flow cytometry analyses indicated that HIV-1-infected CD4^+^ T lymphocytes expressed 1.5–2.0 times more CD59 protein at their cell surface as compared to uninfected cells (Figure 3C). Taken together, these results indicate that HIV-1 infection of CD4^+^ T cells induces the upregulation of CD59 receptor at the mRNA and cell surface protein levels, and this condition appears to be linked to the downregulation of miRNAs, namely miRNAs-21, -26a and 29a, predicted to target CD59.

### 3.3. CD59 Is a Target of miRNA-26a

**Figure 4:**
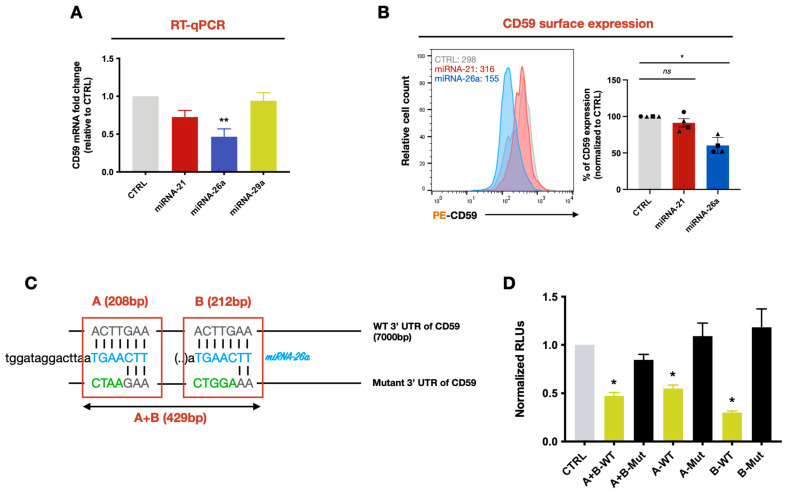
CD59 mRNA is a target of miRNA-26a. (**A**) CD59 mRNA levels were measured in miRNAs-21, -26a and -29a mimics or control-nucleofected primary CD4^+^ T cells via real-time qPCR. Shown is the mean fold-change compared to control transfected cells (in gray, which is set at 1) (n = 4). Error bars represent the SEM. Statistical significance was determined using the nonparametric Kruskal Wallis test, values: ** *p* < 0.01. (**B**) CD59 surface expression was evaluated in miRNAs-21 and -26a mimics or control-nucleofected primary CD4^+^ T cells via flow cytometry. Shown is the cell surface expression of CD59 from CD4^+^ T cells of four blood donors (normalized to CTRL, which was set to 100%) with a representative result on the left (MFI are shown). Error bars represent the SEM. Statistical significance was determined using the nonparametric Kruskal Wallis test, values: * *p* < 0.05. (**C**) The MIR-Report system was used to validate whether the CD59 3′ UTR was a target of miRNA-26a. MiRNA blue nucleotides represent those predicted to interact with the CD59 3′ UTR. WT or mutated (substituted nucleotides in green) fragments of the 3′ UTR encompassing the predicted sequences recognized by miRNA-26a were fused to the F-Luciferase (F-Luc) gene. (**D**) HEK-293T cells were transfected with miRNA-26 mimics, the reporter plasmids with the indicated fragment of the WT (in yellow) or mutated (in black) 3′ UTR and a Renilla-Luciferase (R-Luc) normalizing control. Following cell lysis, relative light units (RLUs) were measured, and F-Luc was normalized to the negative control (in grey, which is set to 1). Error bars represent the SEM. Statistical significance was determined using the nonparametric Kruskal Wallis test, values: * *p* < 0.05.

To evaluate the impact of these miRNAs on the levels of CD59 mRNA and surface protein expression levels, we nucleofected either miRNAs-21, -26a or -29a mimics into primary CD4^+^ T cells. The transfection of miRNA-26a mimics significantly decreased CD59 mRNA levels after 48 h (~2-fold less, *p* = 0.0065) and CD59 protein surface expression 96 h post-nucleofection (approximately 50%, *p* = 0.0285) when compared to controls (Figure 4A,B). Neither miRNA-21 nor miRNA-29a mimics had a significant effect on CD59 mRNA expression levels. Consistently, we confirmed that miRNA-21 mimics had no effect on CD59 surface expression levels (Figure 4A,B). These results suggest that CD59 expression can be downregulated by miRNA-26a but not by miRNAs-21 or -29a in CD4^+^ T cells.

An online database (TargetScanHuman, www.targetscan.org (accessed on 26 January 2024)) analysis predicted that the 3′ UTR of CD59 mRNA could be targeted by miRNA-26a at two potential sites (Figure 4C). To validate whether these sites were targeted by miRNA-26a, we transiently expressed a construct encoding the Firefly Luciferase (F-Luc) gene fused to a 429-base-pair fragment of the 3′ UTR encompassing the two potential target sites (A+B-WT) in HEK-293T cells. These cells were also co-transfected with miRNA-26a or control mimics. F-Luc activity in the cell lysates was significantly reduced in cells transfected with miRNA-26a mimics (~2-fold less, *p* = 0.0338) when compared to the control (Figure 4D). Furthermore, mutations in the CD59 3′ UTR sequences predicted to be targeted by miRNA-26a (Figure 4C, A+B-Mut) prevented the silencing effect on F-Luc (Figure 4D). We also tested, in this system, two different CD59 3′ UTR fragments (A and B) (Figure 4C), each containing one of the two single individual sites targeted by miRNA-26a and found that both sites are functional and independent (Figure 4D). Altogether, these data further validate that miRNA-26a can regulate CD59 expression levels by targeting two specific sites complementary to the miRNA-26a seed sequence located in the 3′ UTR of CD59 mRNA.

### 3.4. CD59 Affects the Susceptibility of HIV-1 to Antibody-Dependent Complement-Mediated Lysis

Given the critical inhibitory regulatory function of CD59 in CML and the fact that its expression is upregulated during HIV-1 infection of CD4^+^ T lymphocytes, we examined the impact of CD59 expression on the susceptibility of HIV-1-infected cells and released virions to ADCML. To this end, we took advantage of a T lymphoblast CEM-CCR5 cell line [54] as a model for the regulation of CD59 by miRNA-26a. Indeed, upon HIV-1 infection of this cell line, we observed the downregulation of miRNA-26a expression (~2-fold less, *p* = 0.0286) and the concomitant upregulation of CD59 mRNA (3.5-fold, *p* = 0.0079) and surface protein levels (Figure S3A). We thus generated two CD59_knock-out (KO) clones of the CEM-CCR5 cell line (CEM-CD59_KO_clone 1 and CEM-CD59_KO_clone 2) using the clustered regularly interspaced short palindromic repeat (CRISPR)/Cas9 technology as described in the Methods. A CEM-CD59_control cell line in which the CRISPR/Cas9 guide sequence was absent was also generated. As shown in Figure S3B, both CEM-CD59_KO clones exhibited a marked reduction in total, as well as cell-surface, CD59 expression levels as compared to the control cell line. Neither cell proliferation nor CD4/CCR5 HIV-1 receptor levels were affected by knocking out CD59 in the CEM-CCR5 cell line (Figure S3C).

We next assessed the susceptibility of these cell lines to ADCML using PGT121, a bNAb that targets a V3-glycan-dependent epitope on HIV-1 gp120 [55], as a model Ab in this system. Control or CEM-CD59_KO cells infected at comparable infection frequencies were exposed to PGT121 in the presence of normal human serum (NHS), as a source of active complement, or in the presence of heat-inactivated human serum (HIHS) as a control. The percentage of dead cells in the GFP-positive cells (based on Zombie NIR fluorescence) was measured via flow cytometry. In a set of two experiments, in the presence of NHS, infected CEM-CD59_KO cells were more susceptible to ADCML as compared to the CEM-CD59_control cells (Figure S4, upper panels). This increased susceptibility of CEM-CD59_KO cells to ADCML was abolished in conditions using HIHS (Figure S4, lower panels).

**Figure 5:**
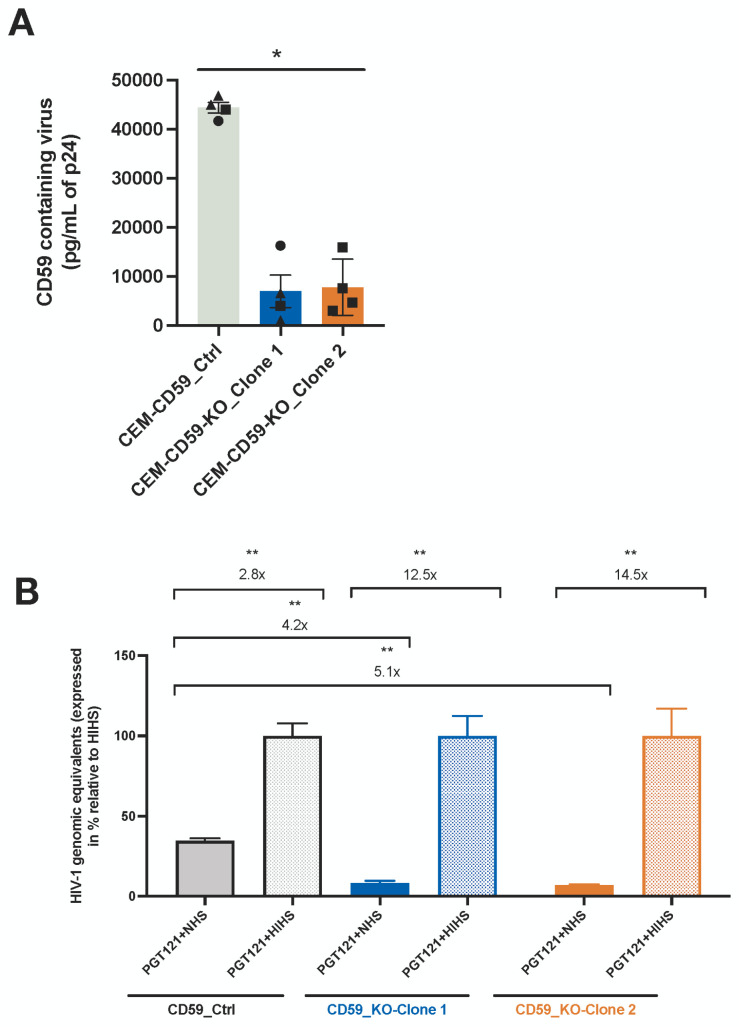
Decreased packaging of CD59 in released virions enhances their susceptibility to ADCML. (**A**) Detection of CD59 incorporation into virions produced from HIV-1-infected CEM-CD59_control or CEM-CD59_KO cells via an Ab capture assay. Equal amounts of virus released from each cell line were analyzed. Data shown indicate the mean levels of CD59-containing virus, as measured via ELISA for p24 (pg/mL), following the lysis of anti-CD59 bead-associated virus (background capture with the isotypic control was subtracted). Error bars represent the SEM. Statistical significance was determined using the nonparametric Mann–Whitney’s test, values: * *p* < 0.05. (**B**) Shown are the normalized total HIV genomic equivalents of PGT121-treated viruses (with either NHS or HIHS) from CEM-CD59_control or CEM-CD59_KO infected cells as measured via real-time qPCR, following the reverse transcription of viral RNA. Values are normalized relative to the mean HIHS condition, which is set at 100%. Error bars represent the SEM. Statistical significance was determined using the nonparametric Mann–Whitney’s test, values: ** *p* < 0.01. In all conditions, equal amounts of virus released from each cell line were analyzed. See also Figures S3 and S4.

Knowing that CD59 is packaged into HIV-1 virions likely as a mean to protect viral particles from ADCML [13], we characterized the impact of CD59 packaging into released virions on their susceptibility to lysis. Using a virion capture-assay allowing for the detection of CD59-containing virions, we confirmed that infected CEM-CD59_KO cell lines produced virions with significantly reduced CD59 levels, as compared to viral particles produced from their control cell line counterpart (~6.5-fold less, *p* = 0.285 for clone 1; ~5-fold less, *p* = 0.0482 for clone 2) (Figure 5A). These results are consistent with the marked reduction in CD59 expression observed at the cell surface of the CEM-CD59_KO cell lines compared to the CEM-CD59_control (Figure S3B). HIV-1 virions released from CEM-CD59_KO and CEM-CD59_control cells were treated with PGT121 and either NHS or HIHS. RNA released from lysed virions was then digested through RNase treatment, and viral RNA from the remaining complement-mediated lysis-resistant intact virions was extracted and quantified via real-time PCR. As shown in Figure 5B, viruses produced from the CEM-CD59_KO cell lines were much more sensitive to ADCML (NHS conditions) than those produced from the control cell line (4.2-fold for clone 1 and 5.1-fold for clone 2, *p* = 0.0022), confirming that the packaging of CD59 into HIV-1 virions protects viruses from ADCML.

### 3.5. Enhanced Levels of miRNA-26a Promote ADCML of HIV-1 by Reducing CD59 Packaging into Released Virions

**Figure 6:**
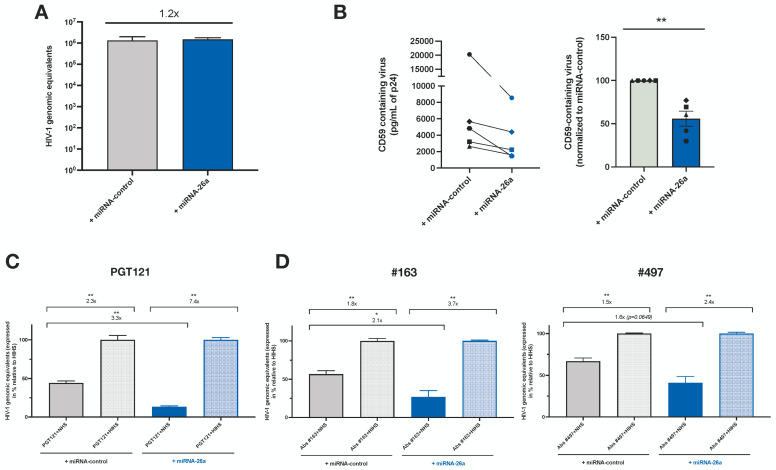
Enhanced expression of miRNA-26a during HIV-1 infection renders released virions more susceptible to ADCML. (**A**) Primary CD4^+^ T cells were nucleofected with miRNA-26a or miRNA-control mimics for 72 h and infected with HIV-1 for 48 h (n = 3). Data shown indicate the total HIV-1 produced (as genomic equivalents of HIV-1) from infected cells, as measured via real-time qPCR, following the reverse transcription of viral RNA. Error bars represent the SEM. (**B**) Detection and quantification of CD59-containing virus particles using an Ab-capture assay of virions produced by infected cells transfected with miRNA-26a or negative control mimics. The left panel indicates the mean level of CD59-containing virus as determined via p24 ELISA (pg/mL) after the lysis of virus captured with CD59-antibody-conjugated beads (the capture assay background obtained with the isotypic Ab control was subtracted). The right panel indicates the mean level of CD59-containing virus normalized to the value obtained with the control mimic condition (set to 100%). In all conditions, equal amounts of virus were analyzed. Error bars represent the SEM. Statistical significance was determined using the nonparametric Mann–Whitney’s test, values: ** *p* < 0.01. (**C**) Shown are the normalized total HIV-1 genomic equivalents of PGT-121-treated viruses from infected cells transfected with either miRNA-26a or negative control mimics, as measured via real-time qPCR, following the reverse transcription of viral RNA. Values are normalized relative to the means of those obtained with HIHS-treated viruses, which were set to 100%. Error bars represent the SEM. Statistical significance was determined using the nonparametric Mann–Whitney’s test, values: ** *p* < 0.01. (**D**) Shown are the normalized total HIV-1 genomic equivalents of Ab-treated viruses from infected cells transfected with miRNA-26a or negative control mimics, as measured via real-time qPCR, following the reverse transcription of viral RNA. Values are normalized relative to the means of those obtained with HIHS-treated viruses, which were set to 100%. Abs were purified from the sera of viremic individuals #163 (left panel) or #497 (right panel). Error bars represent the SEM. Statistical significance was determined using the nonparametric Mann–Whitney’s test, values: * *p* < 0.05, ** *p* < 0.01. In all conditions, equal amounts of virus were analyzed. See also Figure S5.

To specifically examine the impact of augmenting miRNA-26a levels during HIV-1 infection, we nucleofected primary CD4^+^ T cells with miRNA-26a or control mimics and infected them with HIV-1. Figure 6A shows that the transfection of miRNA-26a mimics does not interfere with viral replication as virus production was comparable between miRNA-26a and control mimic-treated cells. Using the virion capture-assay described above, we also confirmed that cells treated with miRNA-26a mimics produced approximately 50% less CD59-containing virions than their control-treated counterparts (~2-fold less, *p* = 0.0079), suggesting that the downregulation of CD59 expression by miRNA-26a leads to the reduced packaging of the protein into released virions (Figure 6B). Importantly, viruses produced from miRNA-26a mimic-treated cells displayed an enhanced susceptibility to PGT-121-dependent CML (NHS conditions) as compared to the control (3.1-fold, *p* = 0.0022) (Figure 6C). These results suggest that a 50% modulation in CD59 expression and incorporation levels into released virus particles is sufficient to effectively affect their sensitivity to ADCML (Figure 4A,B and Figure 6B). To further validate the result obtained with PGT121, we repeated these experiments using purified Abs from the sera of viremic individuals (#163 and #497). We first confirmed that the exposure of virus to purified Abs alone did not induce the lysis of virions (Figure S5A). Viruses produced by cells treated with miRNA-26a mimics displayed an enhanced susceptibility to ADCML in the presence of Abs from sera #163 (2.1-fold, *p* = 0.0260) or #497 (1.6-fold, *p* = 0.0649) as compared to controls (Figure 6D). The results obtained with Abs from serum #163 were further validated using virus produced during the infection of CEM-CD59 KO_clone 1 (Figure S5B); in this context, the virus produced displayed a 4.4-fold increased susceptibility to ADCML as compared to the control (Figure S5B). Taken together, these results show that CD59 expression and packaging into virions during the infection of CD4^+^ T cells can be downregulated by miRNA-26a, a condition that affects the susceptibility of released virions to ADCML. Based on these results, we infer that the downregulation of miRNA-26a by HIV-1 is likely a means to render infected cells and released virions more resistant to ADCML through optimal CD59 expression and packaging into virions.

### 3.6. The Downregulation of miRNA-26a during HIV-1 Infection of CD4^+^ T Lymphocytes Is Contingent on Viral DNA Integration and Expression

**Figure 7:**
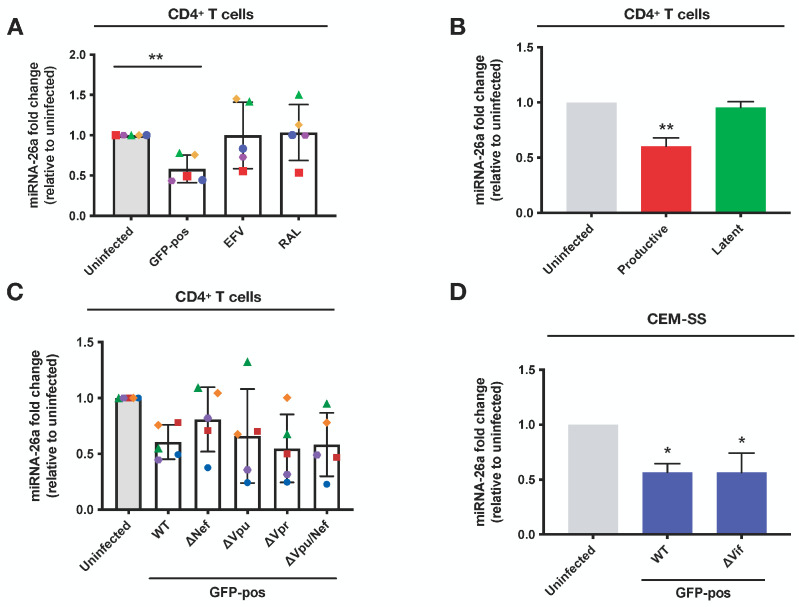
Analyses of HIV-1 determinants regulating miRNA-26a expression during infection. (**A**) Primary CD4^+^ T cells were pretreated with efavirenz (EFV, 10 μM) or raltegravir (RAL, 20 μM) and infected with NL4.3-ADA-GFP (WT), and their total RNA was extracted. MiRNA-26a levels in uninfected, infected GFP-positive and EFV- or RAL-treated cells were measured via real-time qPCR (n = 5). Shown are the mean fold-changes compared to uninfected cells (gray histogram, which is set to 1). Error bars represent the SEM. Statistical significance was determined using the nonparametric Mann–Whitney’s test, values: ** *p* < 0.01. (**B**) Primary CD4^+^ T cells were infected with the dual reporter HIV Nef-2A-CRIMZs viruses, which allows for the distinction between cells harboring LTR-directed active transcription (E2-Crimson-positive/ZsGreen-positive) from cells harboring LTR-directed inactive transcription (E2-Crimson-negative and ZsGreen-positive cells). The different cell populations were sorted, and their total RNA was extracted. MiRNA-26a expression levels in uninfected, productively and latently infected CD4^+^ T cells were measured via real-time qPCR (n = 6). Shown are the mean fold-changes compared to uninfected cells (in gray, which is set at 1). Error bars represent the SEM. Statistical significance was determined using the nonparametric Kruskal Wallis test, values: ** *p* < 0.01. (**C**) Primary CD4^+^ T cells were infected with the indicated mutant NL4.3-ADA-GFP viruses, and their RNA was extracted 48 h post-infection. MiRNA-26a expression in uninfected and infected GFP-positive cells was measured via real-time qPCR (n = 5). Shown are the mean fold-changes compared to uninfected (gray histogram, which is set to 1). Error bars represent the SEM. (**D**) CEM-SS cells were infected with NL4.3-ADA-GFP (WT) or NL4.3-ADA-GFP (ΔVif), and their RNA was extracted 48 h post-infection. MiRNA-26a expression levels in uninfected and infected cells were measured via real-time qPCR (n = 3). Shown are the mean fold-change compared to uninfected (in gray, which is set at 1). Error bars represent the SEM. Statistical significance was determined using the nonparametric Mann–Whitney’s test, values: * *p* < 0.05. See also Figure S6.

We next analyzed the viral determinants that contribute to the downregulation of miRNA-26a expression in CD4^+^ T lymphocytes during HIV-1 infection. To define the step of the infection cycle that is important for miRNA-26a expression downregulation, we treated cells with either the HIV-1 reverse transcriptase inhibitor efavirenz (EFV) or the HIV-1 integrase inhibitor raltegravir (RAL) prior to their infection with HIV-1. As shown in Figure 7A, we observed the downregulation of miRNA-26a in untreated infected GFP-positive sorted cells. However, the treatment of cells with EFV or RAL abolished this downregulation, suggesting that the integration of proviral DNA into the genome of target cells is a necessary step for miRNA-26a downregulation. In order to further validate these results, we used a fully replicative dual reporter virus system, which is very similar to a single-cycle dual reporter virus system that we previously described and characterized [23,35]. This fully infectious and replicative reporter virus system (HIV Nef-2A-CRIMZs) enables cells harboring transcriptionally-inactive proviruses (ZsGreen positive, this reporter gene being under the control of an Elongation factor 1-alpha promoter) to be distinguished from cells that contain specifically transcriptionally active integrated proviruses (both ZsGreen positive and E2-Crimson positive, the later reporter gene being under the control of the HIV-1 LTR). Indeed, we show that following the treatment of infected cells with TNF-alpha as a virus-latency-reversing agent, the frequency of infected cells harboring a transcriptionally inactive provirus (only ZsGreen positive; latent) was reduced, and a concomitant increase in the frequency of infected cells with transcriptionally active integrated provirus (both E2-Crimson/ZsGreen positive; productive) was detected (Figure S6A). Additionally, productively infected cells, which are predicted to express Nef and Vpu, were found to display, as expected, the downregulation of the CD4 receptor, a condition not observed in bystander cells or latently infected cells (Figure S6B).

Analyses of miRNA expression in the different isolated populations of infected lymphocytes revealed that miRNA-26a expression levels were decreased in productively infected cells (~1.7-fold less, *p* = 0.0015) but not in latently infected cells (Figure 7B). These results suggest that HIV-1 gene expression is necessary for the downregulation of miRNA-26a during infection. Given that HIV-1 accessory proteins, such as Vpu, have been reported to modulate the expression of miRNAs [45,46], we tested if their expression could impact miRNA-26a levels. To this end, CD4^+^ T lymphocytes were infected with well-established and well-characterized GFP-marked HIV-1 that is isogenic except for the expression of Nef, Vpu, Nef and Vpu or Vpr [31,32,33]. As shown in Figure 7C, expression levels of miRNA-26 were downregulated in cells infected with the different mutant HIV-1 viruses to a comparable extent to those in cells infected with WT HIV-1, suggesting that a reduction in miRNA-26a expression during HIV-1 infection appears to be independent of Vpr, Vpu or/and Nef expression. Given the important role of the Vif accessory protein in counteracting the potent restriction mediated by APOBEC3F/G on HIV-1 reverse transcription [56], we evaluated the effect of Vif on miRNA-26a expression in the CEM-SS cell line, which does not express APOBEC3F/G and is permissive to infection by Vif-defective viruses [57]. MiRNA-26a expression was found to be downregulated to the same extent in CEM-SS cells infected with WT virus (~1.8-fold less, *p* = 0.0500) or Vif-deficient virus (~1.8-fold less, *p* = 0.0500), suggesting that the reduction in miRNA-26a expression in HIV-1-infected cells is independent of Vif expression (Figure 7D). Taken together, our results indicate that while the downregulation of miRNA26a during HIV-1 infection requires viral gene expression, this regulation does not appear to be dependent on HIV-1 accessory gene expression.

## 4. Discussion

In this study, we performed RNA-seq of miRNAs and mRNAs in HIV-1-infected CD4^+^ T cells and further analyzed their expression profiles relative to uninfected cells (Figure 1). Since the miRNA and mRNA profiling was conducted simultaneously on the same sorted infected (GFP-positive) and uninfected cells, as a baseline reference, this allowed us to establish potential links between the degree of expression of specific candidate cellular genes and putative targeting miRNAs. We found that the infected cell population displayed important changes in the cellular transcriptomes and miRNA expression profiles in relation to the uninfected population. Among the 222 cellular genes for which expression was either upregulated (188) or downregulated (34) (Figure S2), we identified, through a systematic literature search, several candidates that could have either positive or negative effects on HIV-1 replication or persistence (Figure 2A). To assess whether differentially expressed genes in the infected population could be regulated by miRNAs, we cross-analyzed the most differentially expressed miRNAs and found that three of the most downregulated miRNAs, namely miRNAs-29a, -21 and 26a, were predicted to target CD59 transcripts, which indeed were upregulated in infected cells (Figure 2, Figure 3 and Figure S2). CD59 is a cell surface protein receptor that plays a negative regulatory role in the complement pathway and is incorporated into budding viruses, a condition that protects HIV-1 particles from ADCML [13]. These findings raised the possibility that HIV-1 is downmodulating the expression of these miRNAs to optimize the expression and packaging of CD59 as a mean to limit the activity of the host complement lysis pathway.

MiRNAs have been shown to play a key role not only in regulating target cell susceptibility to HIV-1 infection but also in promoting viral persistence [19]. Of the three miRNAs that were predicted to target CD59, further validation revealed that miRNA-26a was the only one that could target and regulate CD59 expression in CD4^+^ T cells (Figure 4). MiRNA-26a is a member of the miRNA-26 family, which includes miRNA-26a and miRNA-26b. This family has previously been described to be involved in the regulation of muscle development [58] and glucose metabolism [59], but surprisingly, little is known about its role in immune cell functions or innate immunity. Recently, a study showed that the miRNA-26 family plays a role in the early development and transformation of B lymphocytes, particularly in the differentiation of pre-B cells, although the mechanism behind this phenomenon is not fully elucidated [60].

Our results suggest that miRNA-26a regulates the expression of CD59 in CD4^+^ T lymphocytes and that its downregulation during HIV-1 infection leads to the upregulation of CD59 mRNA levels and surface protein expression (Figure 3 and Figure 4), conditions that are likely to increase its packaging into released virus particles. Knowing that CD59 incorporation into viral particles protects HIV-1 from CML activity, our results suggest an important role of miRNA-26a in regulating the susceptibility of HIV-1 to CML (Figure 5 and Figure 6). Since the packaging of CD59 is not an exclusive property of HIV-1 as it has also been shown in other enveloped viruses [61,62], namely human T lymphotropic virus type-1 (HTLV-1), human cytomegalovirus (HCMV) and hepatitis virus type C (HCV), it would be interesting to assess whether these viruses promote CD59 packaging by downmodulating miRNA-26a during infection. It is important to note that the functional results of this study were obtained using transfected mimics of miRNA-26a. It remains unclear whether the physiological levels of endogenous miRNA-26a, which are likely lower than the concentration of mimics, are sufficient to exert similar effects.

Our results show that miRNA-26a expression levels are decreased in CD4^+^ T cells upon HIV-1 infection, and we provide evidence that this reduction requires the integration of the viral DNA into the genome of target cell, as well as the expression of HIV-1 genes (Figure 7). However, expression of the accessory proteins Vif, Vpr, Nef and Vpu does not appear to mediate the downregulation of miRNA-26a, as viruses deficient for the expression of these viral proteins were still competent in downregulating miRNA-26a expression. These results suggest that other viral proteins, such as Tat, Rev, the Env glycoproteins or the products of the Gag and Gag-pol precursors, might potentially be involved. Alternatively, HIV-1 may indirectly induce a cellular response that regulates miRNA-26a expression. Little is known about the direct regulators of miRNA-26a expression, although previous reports have shown a double negative feedback loop between miRNA-26a and the negative transcriptional regulators EZH2 [63] and E2F7 [64].

HIV-1 is protected from CML via the incorporation of several complement-regulatory proteins, such as CD46, CD55 and CD59, into viral particles [12]. CD59 controls the formation of the MAC at the terminal stage of the complement activation common to all known activation pathways [11], while CD55 prevents the formation of C3 convertases, and C5 [65] and CD46 act as inactivators of C3b and C4b [66]. In this study, we provide evidence supporting the notion that the HIV-1-mediated enhancement of CD59 cell-surface expression and packaging helps protect virions and infected cells from ADCML. Indeed, we show that knocking down CD59 via CRISPR-Cas9 in target CD4^+^ T cells enhances the susceptibility of cells and virions to ADCML. Although our data on HIV-1-infected CEM-CD59_control or CEM-CD59_KO cells (Figure S4) may require more substantial analyses, particularly using several other anti-HIV-1 antibodies, these findings are collectively consistent with results obtained previously using the Ab-mediated blockade of CD59 [67,68], as well as evidence that GPl-anchor deficiency, which leads to an absence of CD59 at the cell surface, renders released HIV-1 susceptible to complement attack [69]. Taken together, these results suggest that limiting CD59 expression or targeting its function at the cell surface of infected cells could represent an approach to restore optimal complement activation and increase the efficiency of Ab-mediated responses against HIV-1.

The ability to activate complement in response to HIV-1 infection is antibody-dependent. Indeed, Dufloo et al. showed that polyclonal Abs present in the sera of infected individuals trigger complement activation less effectively than bNAbs [70]. If the concentrations of the patient-derived antibodies at the dilutions used in our studies are comparable to that of PGT121 in our experiments, our observations are consistent with their results. Indeed, we show a much greater ADCML effect on virions exposed to the bNAb PGT121 as compared to a pool of Abs purified from the sera of viremic individuals, which most likely target diverse epitopes (Figure 6). Furthermore, it is likely that the neutralizing activity of Abs present in the viremic patient sera tested is less significant than that of bNAbs, of which development takes place late in the course of infection and only in a limited number of individuals [71]. Given that complement is activated by Ab hexamers assembled on the surface via Fc–Fc interactions [72], it is likely that the orientation of the Fc region and the Ab density are critical factors for complement activation by a given bNAb. Indeed, Abs targeting the CD4-binding site or the V3 loop can bind up to three Fab fragments on an Env trimer, which would promote Fc–Fc interactions [73]. Altogether, these points could explain why the more efficient ADCML of virions was observed using PGT121 as compared to Abs purified from infected individual sera and could also explain the variability detected using Abs from the sera of the two viremic individuals (Figure 6).

In conclusion, our study identifies miRNA-26a as a post-transcriptional negative regulator of CD59 expression in CD4^+^ T cells. Our results show that HIV-1 reduces the level of this miRNA during infection likely via the expression of a viral protein for which the identity remains to be defined. We also provide evidence that the addition of exogenous miRNA-26a mimics to infected cells reduces the resistance of HIV-1 to ADCML. This miRNA is yet another example of a miRNA of which the modulation of expression allows HIV-1 to escape the host immune response and more specifically ADCML. This mechanism could be targeted to augment the potency of antibody-based preventive or therapeutic strategies against HIV-1.

## Data Availability

The online GEO accession numbers for the reported mRNA-seq and miRNA-seq data are GSE247191 (mRNA-seq) and GSE247194 (miRNA-seq), respectively.

## Supplementary Materials

The following are available online at https://www.mdpi.com/article/10.3390/v16071076/s1. There are Supplementary figures (Figures S1–S5) and a supplementary Table S1 related to this work: Figure S1. Characterization of the infected cell populations used for the RNA-seq, related to Figure 1. Figure S2. List for genes for which mRNA expression levels are modulated in the GFP-positive population compared to the uninfected group, related to Figure 1. Figure S3. Modulation of miRNA-26a and CD59 expression levels in HIV-1-infected CEM-CCR5 cells and characterization of CEM-CD59_KO cells, related to Figure 5. Figure S4. Reduction of CD59 expression in HIV-1-infected cells enhances their susceptibility to ADCML, related to Figure 5. Figure S5. ADCML assay with antibodies purified from the sera of viremic individuals, related to Figure 6. Figure S6. Characterization of CD4^+^ T cell subpopulations following infection with the dual reporter HIV Nef-2A-CRIMZs virus, related to Figure 7. Table S1. Sequences of miRNA mimics and oligonucleotides used in this study.
