# Supplementary material for: Downregulation of miRNA-26a by HIV-1 Enhances CD59 Expression and Packaging, Impacting Virus Susceptibility to Antibody-Dependent Complement-Mediated Lysis
Source: Viruses. 2024 Jul 4;16(7):1076. doi: 10.3390/v16071076 (PMC11281366; doi:10.3390/v16071076)
Supplement: Supplementary file 1 [file viruses-16-01076-s001.zip › viruses-3066144-supplementary.pdf]

SUPPLEMENTARY FIGURES

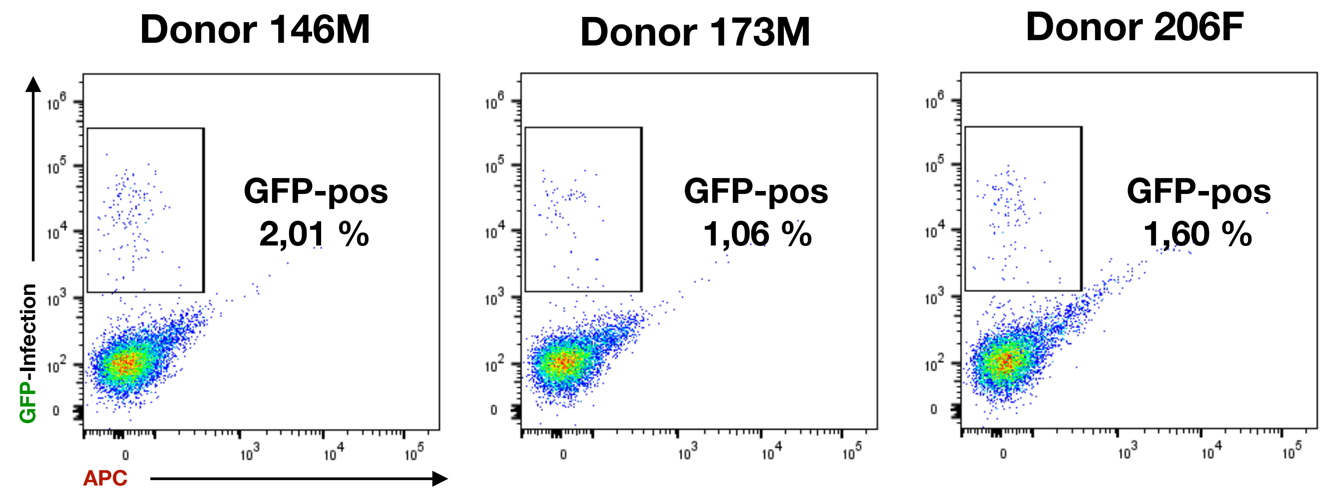

Figure S1. Characterization of the infected cell populations used for the RNA-seq, related to Figure 1.
Primary $CD4^{+}$ T-cells from three different donors were isolated, activated and then infected with NL4.3-ADA-GFP (WT). Frequency (%) of infected cells was determined by measuring GFP expression using flow cytometry.

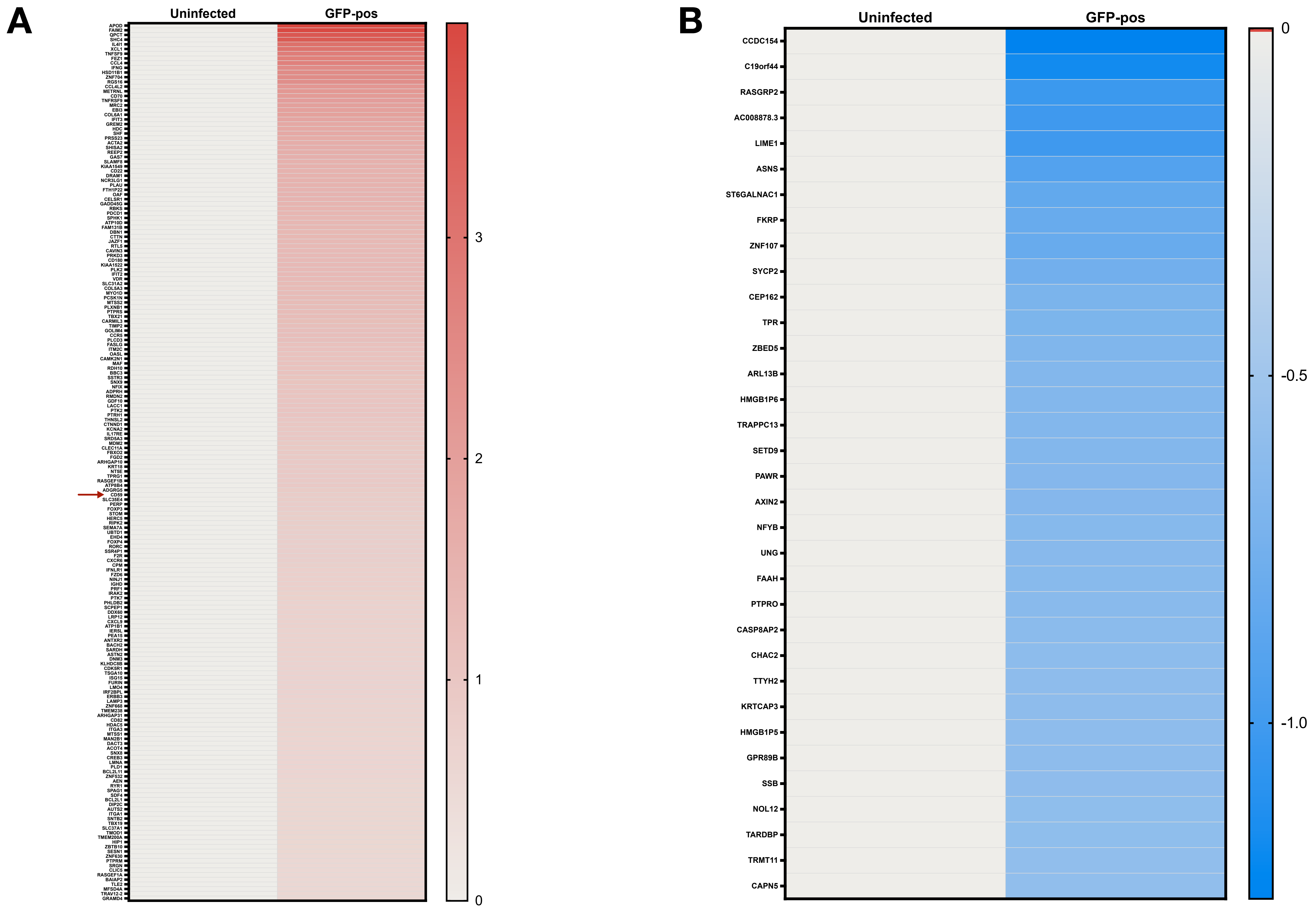

Figure S2. List for genes for which mRNA expression levels are modulated in the GFP-positive population compared to uninfected, related to Figure 1.

(A) The heatmap depicts the 188 mRNAs specifically upregulated in the GFP-positive population. CD59 is identified with a red arrow. (B) The heatmap depicts the 34 mRNAs specifically downregulated in the GFP-positive population. In (A) and (B), the scale bar is in log2(Fc).

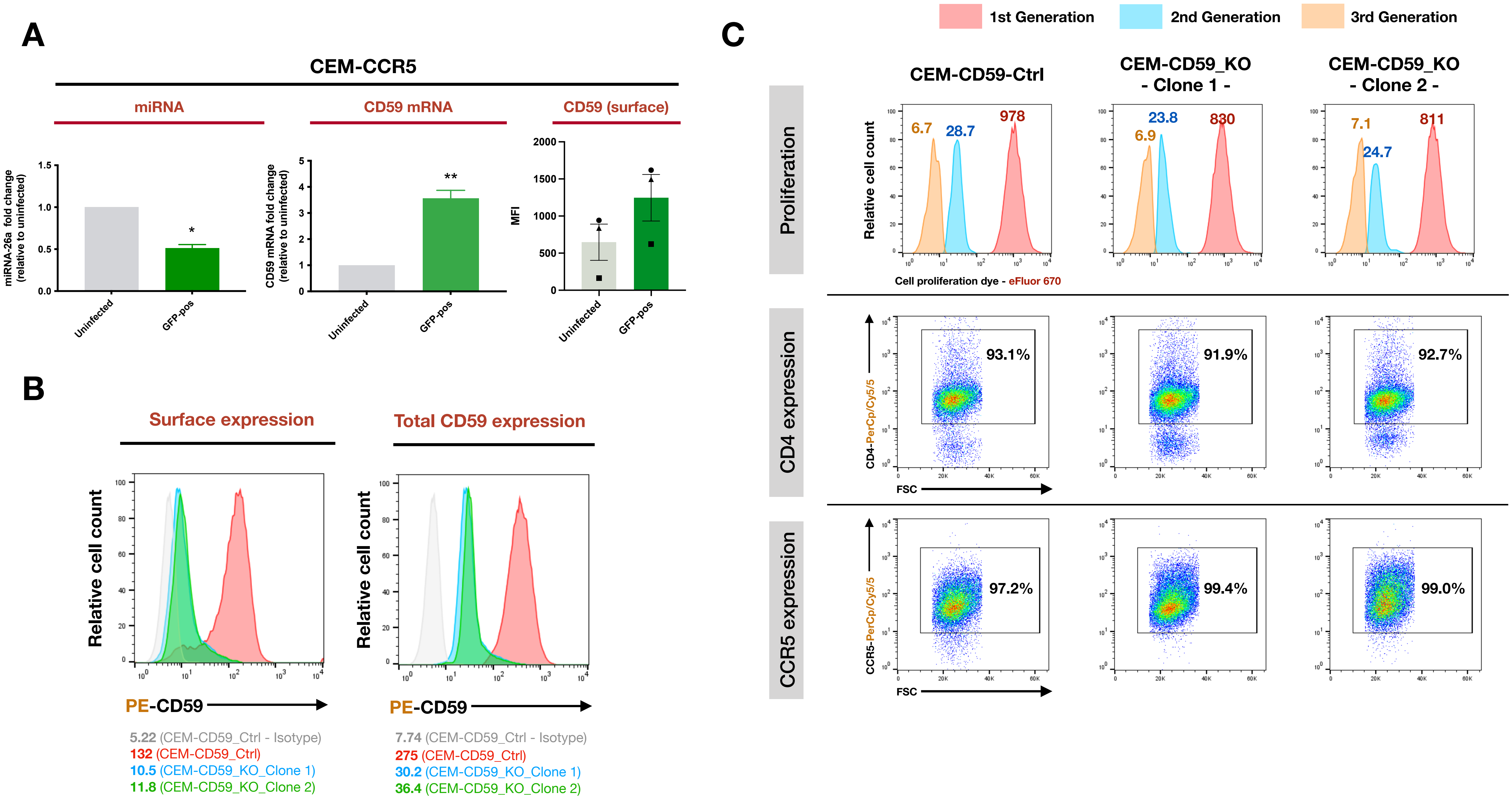

Figure S3. Modulation of miRNA-26a and CD59 expression levels in HIV-1 infected CEM-CCR5 cells and characterization of CEM-CD59_KO cells, related to Figure 5.

(A) MiRNA-26a and CD59 mRNA levels in uninfected and HIV-1 infected CEM-CCR5 (GFP-positive) (n=4) were measured by real-time qPCR. Shown are the mean fold changes compared to uninfected (in gray, which is set at 1.0). Cell-surface CD59 expression levels were evaluated by flow cytometry. the MFI was compared between uninfected and infected CEM-CCR5 (n=2). Error bars represent SEM. Statistical significance was determined by Mann-Whitney's test, values: *p < 0.05, ***p* < 0.01. (B) Cell-surface and total CD59 expression levels were determined by flow cytometry. The MFI values are indicated for the CEM-CD59_control and CEM-CD59_KO cell lines. Shown is one representative experiment (n=3). (C) Cell proliferation (eFluor-670) and frequency of cells expressing CD4 (PerCp/Cy5.5) or CCR5 (PerCp/Cy5.5) were measured by flow cytometry in the CEM-CCR5 control cell line and two different CEM-CD59_KO lines. Shown is a representative experiment (n=2).

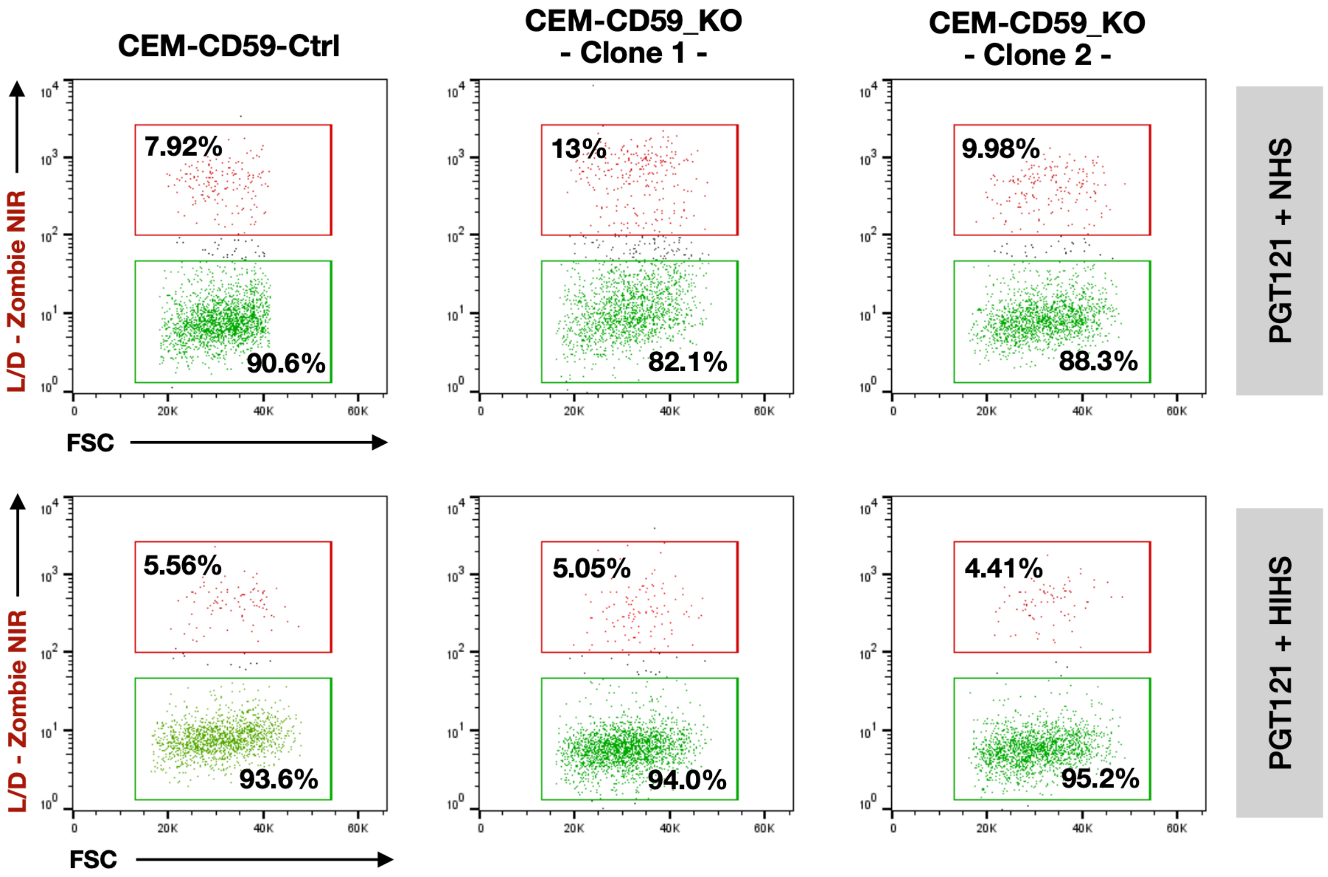

Figure S4. Reduction of CD59 expression in HIV-1 infected cells enhance their susceptibility to ADCML, related to Figure 5.
The level of dead cells in GFP-positive populations was measured by flow cytometry and compared between CEM-CD59_Control or CEM-CD59_KO cells exposed to the indicated conditions (PGT121 + normal human serum (NHS) and PGT121 + heat-inactivated human serum (HIHS)). Shown are data for one representative experiment (n=2).

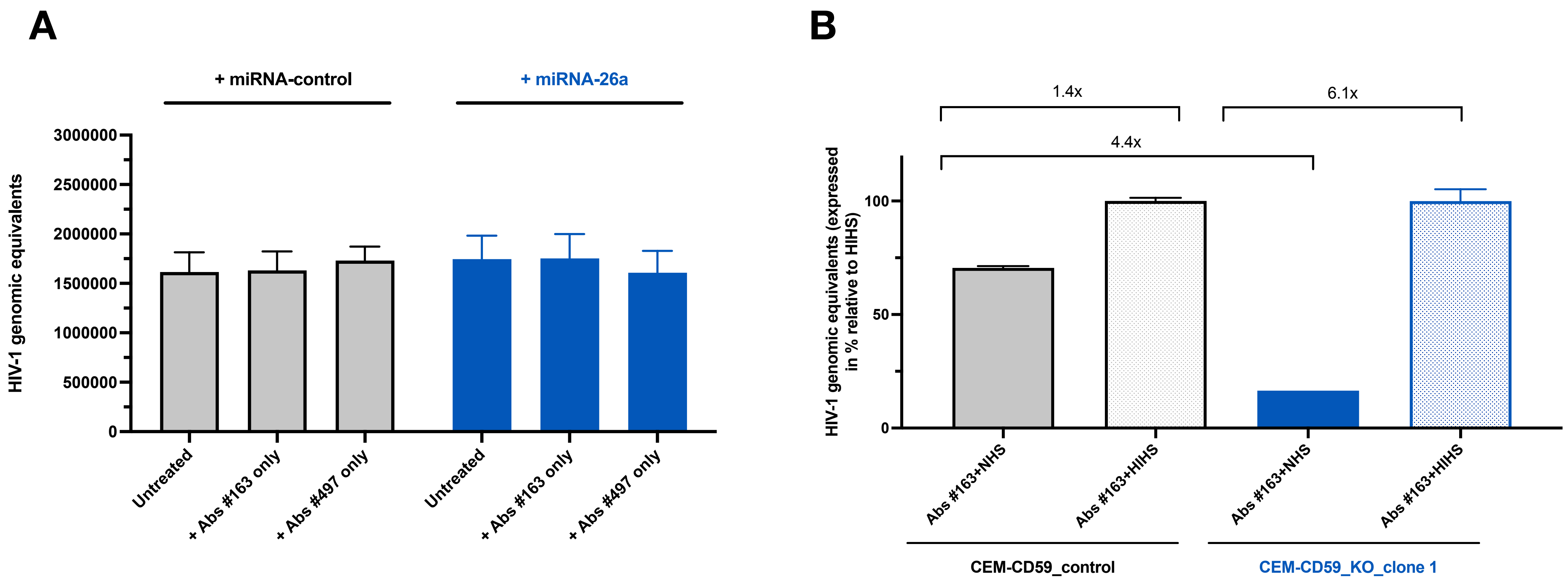

Figure S5. ADCML assay with antibodies purified from the sera of viremic individuals, related to Figure 6.
(A) Shown are the total HIV-1 genomic equivalents of viruses from infected cells transfected with miRNA-26a or negative control mimics measured by real-time qPCR, following reverse transcription of viral RNA. Viruses were treated (or not) only with the indicated Abs purified from the sera of viremic patients, to control for non-specific lysis. Error bars represent SEM. (B) Shown are the normalized genomic equivalents HIV-1 of Ab#163-treated viruses from CEM-CD59_control or CEM-CD59_KO_clone 1-infected cells in the presence of NHS or HIHS. Values are normalized relative to the mean of those obtained with HIHS-treated viruses, which were set at 100%. Error bars represent SEM (n=2).

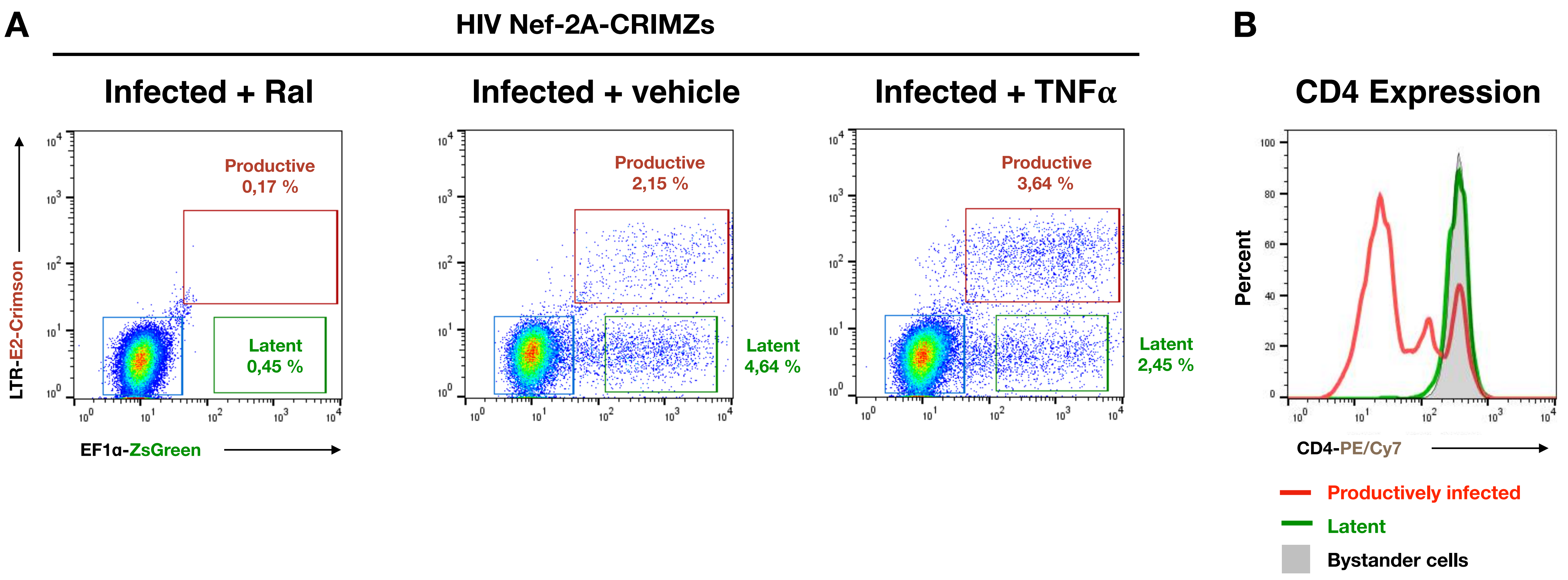

Figure S6. Characterization of CD4+ T cell subpopulations following infection with the dual reporter HIV Nef-2A-CRIMZs virus, related to Figure 7. (A) CEM-CCR5 cells were infected with HIV Nef-2A-CRIMZs for two days in the presence of Raltegravir treatment or its absence (see vehicle control panel). Cells were reactivated after 24h of infection with TNF-alpha (10ng/mL) or the vehicle control for one day. The frequency of productively and latently HIV-1 infected cells was determined by flow cytometry based on E2-Crimson and ZsGreen expression 48h after infection. Productively infected cells are defined as E2-Crimson-positive/ZsGreen-positive while latently-infected cells are identified as E2-Crimson-negative/ZsGreen-positive. Shown is data from a representative experiment. (B) CD4 expression was analyzed by flow cytometry in bystander cells as well as in latently and productively HIV-1 infected cell populations. Shown is data from a representative experiment.

Table S1 – Sequences of miRNAs mimics and oligonucleotides used in this study

miRNAs mimics

Name	Sequence
Control mimic	UCACCGGGUGUAAAUCAGCUUG
miRNA-21-5p mimic	UAGCUUAUCAGACUGAUGUUGA
miRNA-26-5p mimic	UUCAAGUAAUCCAGGAUAGGCU
miRNA-29a-3p mimic	UAGCACCAUCUGAAAUCGGUUA

General qPCR

Target	Name	Sequence
GAPDH	GAPDH - Reverse	TTGACGGTGCCATGGAATTT
	GAPDH - Forward	GCCATCAATGACCCCTTCAT
snRNA-U6	snRNA-U6 - RT	GTCGTATCCAGTGCAGGGTCCGAGGTATTCGCACTGGATACGA CCAAAAATATG
	snRNA-U6 - Reverse	CAGTGCAGGGTCCGAGGTAT
	snRNA-U6 - Forward	GCGCGTCGTGAAGCGTTC
miRNA-21	miRNA-21 - RT	TAAGCTAGATATGTGAGACGTACGTTG AGTACGTCAAGTCAAGGTCAACAT
	miRNA-21 - Reverse	GGGGTAGCTTATCAGACTGATGTTGA
	miRNA-21 - Forward	TAAGCTAGATATGTGAGACGTACGTTGAGT
miR-26a	miR-26a - RT	ACTTGAAGATATGTGAGACGTACGTTGAGTACGTCAAGTGAAGT CCTATCC
	miR-26a - Reverse	GGGTTCAAGTAATCCAGGATAGGCT
	miR-26a - Forward	ACTTGAAGATATGTGAGACGTACGTTGAG
miR-29a	miR-29a - RT	GGTGCTAGATATGTGAGACGTACGTTGAGTACGTCAAGTGAAGT CTAACCGA
	miR-29a - Reverse	GGGTAGCACCATCTGAAATCGGTTA
	miR-29a - Forward	GGTGCTAGATATGTGAGACGTACGTTGA
CD59	CD59 - Reverse	TACACTTGTAACCCAGCTTTGG
	CD59 - Forward	GCCAGTCTTTAGCACCAGTTG

HIV-1 qPCR

Target	Name	Sequence
HIV-TOT	ULF1 (1st PCR)	ATGCCACGTAAGCGAAACTCTGGGTCTCTCTGGTTAGAC
	UR1 (1st PCR)	CCATCTCTCTCCTTCTAGC
	LambdaT (2nd PCR)	ATGCCACGTAAGCGAAACT
	UR2 (2nd PCR)	CTGAGGGATCTCTAGTTACC
	HIV-tot probe	/56-FAM/CACTCAAGG/ZEN/CAAGCTTTATTGAGGC/3IABkFQ/

miR-Report assay

Target	Name	Sequence
3' UTR CD59 (Fragment A)	3'UTR CD59-A Forward	GGACTAGTCCTCAACACCAGGAGAGCTTCTCCCAA
	3'UTR CD59-A Reverse (WT)	CCGACGCGTCGGACTTCTTCCTTCAAGTGGGGCTTCCC
	3'UTR CD59-A Reverse (Mut)	CCGACGCGTCGGACTTCTTCCTTCTTAGGGGGCTTCCC
3' UTR CD59 (Fragment B)	3'UTR CD59-B Forward (WT)	GGACTAGTCCTGAAGTAGGTGTGACTTGAACTAGATTGCATGC
	3'UTR CD59-B Forward (Mut)	GGACTAGTCCTGAAGTAGGTGTGCTGGAAACTAGATTGCATG
	3'UTR CD59-B Reverse	CCGACGCGTCGGGTTTATGAAAGCGTTCCATGTGAGAGAGGATG
3' UTR CD59 (Fragment AB)	3' UTR CD59-AB Forward	GGACTAGTCCTCAACACCAGGAGAGCTTCTCCCAA
	3' UTR CD59-AB Reverse	CCGACGCGTCGGGTTTATGAAAGCGTTCCATGTGAGAGAGGATG

CRISPR

Target	Name	Sequence
CD59 CRISPR	guideRNA - Forward	CACCGCAAGGAGGGTCTGTCCTGTT
	guideRNA - Reverse	AAACAACAGGACAGACCCTCCTTGC

HIV Nef-2A-CRIMZs

Target	Name	Sequence
Hi.Fate construct	Hi.Fate-E2-Crimson-EF1a-Zs-Green-OligoA	TGCACGCGTGGAGGGGGCGGTATGGATAGCACTGAGAACG
	Hi.Fate-E2-Crimson-EF1a-Zs-Green-OligoB	GCTACCCGGGTCAGGGCAAGGCGGAGCCGGAGGCG
